# Characteristics of prescriptions and costs for acute upper respiratory tract infections in Chinese outpatient pediatric patients: a nationwide cross-sectional study

**DOI:** 10.1186/s12906-020-03141-w

**Published:** 2020-11-16

**Authors:** Shan Wang, Lihua Liu, Jianchao Liu, Likun Miao, Qian Zhuang, Ning Guo, Jing Zhao, Quanzheng Li, Guoquan Ren

**Affiliations:** 1 Chinese People’s Liberation Army General Hospital, 28 Fuxing Road, Haidian, Beijing, 100853 China; 2 China National Engineering Laboratory of Application Technology in Medical Big Data, 28 Fuxing Road, Haidian, Beijing, 100853 China; 3 National Health Commission of the People’s Republic of China, National Health Development Research Center, Building B3, 9 Chegongzhuang Street, Xicheng, Beijing, 100044 China; 4 Massachusetts General Hospital, Center for Advanced Medical Computing and Analysis, 55 Fruit Street, Boston, Massachusetts 02114 USA; 5 Peking University, Center for Data Science in Health and Medicine, 38 Xueyuan Road, Haidian, Beijing, 100191 China

**Keywords:** Upper respiratory tract infections, Prescription, Cost, Pediatric, Outpatient, China

## Abstract

**Background:**

To understand the characteristics of prescriptions and costs in pediatric patients with acute upper respiratory infections (AURI) is important for the regulation of outpatient care and reimbursement policy. This study aims to provide evidence on these issues that was in short supply.

**Methods:**

We conducted a retrospective cross-sectional study based on data from National Engineering Laboratory of Application Technology in Medical Big Data. All outpatient pediatric patients aged 0–14 years with an uncomplicated AURI from 1 January 2015 to 31 December 2017 in 138 hospitals across the country were included. We reported characteristics of patients, the average number of medications prescribed per encounter, the categories of medication used and their percentages, the cost per visit and prescription costs of drugs. For these measurements, discrepancies among diverse groups of age, regions, insurance types, and AURI categories were compared. Kruskal-Wallis nonparametric test and Student-Newman-Keuls test were performed to identify differences among subgroups. A multinomial logistic regression was conducted to examine the independent effects of those factors on the prescribing behavior.

**Results:**

A total of 1,002,687 clinical records with 2,682,118 prescriptions were collected and analyzed. The average number of drugs prescribed per encounter was 2.8. The most frequently prescribed medication was Chinese traditional patent medicines (CTPM) (36.5% of overall prescriptions) followed by antibiotics (18.1%). It showed a preference of CPTM over conventional medicines. The median cost per visit was 17.91 USD. The median drug cost per visit was 13.84 USD. The expenditures of antibiotics and CTPM per visit (6.05 USD and 5.87 USD) were among the three highest categories of drugs. The percentage of out-of-pocket patients reached 65.9%. Disparities were showed among subgroups of different ages, regions, and insurance types.

**Conclusions:**

The high volume of CPTM usage is the typical feature in outpatient care of AURI pediatric patients in China. The rational and cost-effective use of CPTM and antibiotics still faces challenges. The reimbursement for child AURI cases needs to be enhanced.

**Supplementary Information:**

The online version contains supplementary material available at 10.1186/s12906-020-03141-w.

## Background

Pediatric outpatient visits took up 9.8% of total outpatient visits in China in 2017, among which acute upper respiratory tract infection (AURI) is the first common cause of sicknesses, accounting for more than 60% of total outpatient visits [1]. The primary strategy for the management of AURI is medication therapy. As children are highly vulnerable to irrational drug use, the study of prescriptions in children with AURI is of great importance. There are also financial concerns for pediatric outpatient cases in consideration of its high incidence and vast price gaps in the pharmaceutical market. Evidence-based cost analysis is also essential for payment and reimbursement policy. However, the available research for prescription patterns and its relevant spending in the Chinese pediatric population with AURI remained inadequate. The existing published studies were usually limited to particular facilities or regions, selected insufficient samples, or non-English language. Thus, the results of the studies appeared large discrepancies. For example, the reported usage of antibiotics ranged widely from less than 10% to more than 80% [2–12]. And studies using a nationwide sample have not been found yet. Moreover, much attention was paid to antibiotic usage, but less was concentrated on other types of medicine used. The insufficient evidence made it hard to monitor and regulate outpatient services resulting in the lag of policy adjustment regarding prescribing behavior as well as reimbursement strategies [13–15].

In the present study, we aim to implement a comprehensive investigation into the characteristics of prescriptions and spending of pediatric patients diagnosed with AURI in outpatient sectors. We obtained a large sample of outpatient cases with individual-level clinical information covering a considerable portion of public hospitals across the country. The study tends to supply reliable evidence on this issue. Also, it is the first attempt to use patient-level information on such a scale to analyze pediatric outpatient care in China.

## Methods

### Data source and study design

The data source was from National Engineering Laboratory of Application Technology in Medical Big Data founded by China Development and Reform Commission in 2017. This big data center is dedicated to routinely collecting comprehensive inpatient and outpatient clinical detailed information across a considerable portion of public hospitals in the country. We obtained data from one of the databases called MIPS (military-hospital public services) in the center. It included complete public cases in all military hospitals that provided healthcare services to the public. The unique advantage of the database is that it encompasses large hospital samples which apply identical hospital information system so that their clinical records can be aggregated directly without technical barriers.

We conducted a retrospective cross-sectional study of pediatric patients diagnosed with uncomplicated AURI who visited hospital ambulatory departments, including emergency departments from 1 January 2015 to 31 December 2017. One hundred thirty-eight public tertiary hospitals from 111 cities in 31 provinces (all provinces in mainland China) were covered. All the records were de-identified before analysis so that confidentiality was assured.

### Patients selection and data processing

We included all pediatric patients aged 0–14 years. Patients were identified according to the J00-J06 code section representing all types of AURI diagnoses in the 10th revision of the International Statistical Classification of Diseases and Related Health Problems (ICD-10, Chinese 2012 edition) [16]. For those diagnoses without code in the records, we employed the Natural Language Processing (NLP) technique [17] to standardize the information to identify the AURI patients. The original diagnosis entries were distinguished via the keywords and automatically classified and standardized once matched. The prescriptions also went through the process of standardization by NLP. Original names of medications on prescriptions were distinguished based on China Pharmacopeia (2015 edition) (see more details of NLP standardization process in Additional file 1) [18].

### Data analysis

In our analysis, patients were divided into four age groups as ≤28 days, 29 days to less than 1 year, 1–4 years, and 5–14 years. Hospitals were sorted into four economic regions, namely Northeast, East, Central, and West region, according to the National Bureau of Statistics [19]. We also classified four consultation types in terms of the physician’s professional title and the department where consultations took place. If a physician with a senior professional title provided consultation in the ambulatory department or emergency department, we categorized the case into “expert consultation (EC)” or “expert consultation in the emergency department (EDEC)”, respectively. Otherwise, we sorted the consultation into “general consultation (GC)” or “general consultation in the emergency department (EDGC)”. Health insurances of patients were classified into seven groups including Urban Resident Basic Medical Insurance (URBMI), New Rural Cooperative Medical System (NRCMS), Commercial Health Insurance (CHI), Welfare Health Care (WHC), Preferential Health Care (PHC), Out-of-pocket Payment (OOP) and Others (see the brief introduction of health insurance and payment system of China in Additional file 1). URBMI and NRCMS are two of the three national fundamental social medical insurance schemes in China [20]. CHI included all kinds of commercial health insurances supplemented to social health insurance. WHC refers to the healthcare welfare, which is mostly provided to national civil servants and staff of large state-owned enterprises at the stage. PHC beneficiaries are usually dependents of military personnel and other prior groups who meet the conditions that can enjoy exclusive medical benefits decided by the hospitals. OOP means the patients pay by themselves. “Others” refers to the remaining situations.

We performed a descriptive analysis using SAS software, Version 9.4 (SAS Institute. Inc., North Carolina, USA). We reported characteristics of pediatric patients diagnosed with AURI, the average number of medications prescribed per encounter, the categories of medication used and their percentages, the cost per visit and prescription costs of different categories of drugs. For some of these measurements, we compared differences among subgroups by different ages, regions, insurance types, consultation types, and AURI classifications. Due to the abnormal distribution of costs and numbers of drugs, we employed the Kruskal-Wallis nonparametric test to evaluate differences among subgroups. Student-Newman-Keuls tests were then used to do further pairwise comparisons between any two means. We also performed a multinomial logistic regression to examine the independent effects of these factors on the prescribing behavior in terms of the two commonest prescribed drugs, antibiotics and Chinese medicines.

In our calculation, one encounter stood for an event of a particular patient visiting a specific hospital on a particular day. Multiple visits of the same patient in the same hospital within 7 days were counted as one encounter according to the typical duration of AURI. The expenditure per case was calculated by adding up the charges of each item based on fee-for-service payment. We have transferred Chinese Yuan (CNY) into US dollar (USD) to make it comparable to other geographical settings. The exchange rate used was 1 USD equaled to 6.7 CNY according to the average rate in 2017.

## Results

### Characteristics of pediatric patients diagnosed with AURI

Between Jan 1, 2015, and Dec 31, 2017, the total volume of outpatient visits of children diagnosed with AURI without comorbidities and complexities was 1,002,687 in studied hospitals. The classification of ‘unspecified AURI (J06) ’ had the most cases, followed by acute tonsillitis. The volume of visits each year remains stable, with less than 10% fluctuate (Table 1).

**Table 1:** Characteristics of outpatient visits of pediatric AURI patients in 2015–2017

Characteristics	Total		2015		2016		2017	
	(***N*** = 1,002,687)		(***N*** = 326,617)		(***N*** = 357,366)		(***N*** = 318,704)	
**Sex (n, %)**								
Male	556,797	(55.5%)	182,885	(56.0%)	198,389	(55.5%)	175,523	(55.1%)
Female	445,890	(44.5%)	143,732	(44.0%)	158,977	(44.5%)	143,181	(44.9%)
**Age (n, %)**								
≤ 28 days	1503	(0.1%)	469	(0.1%)	575	(0.2%)	459	(0.1%)
> 28 days and < 1 year	78,274	(7.8%)	29,428	(9.0%)	24,848	(7.0%)	23,998	(7.5%)
1–4 years old	509,471	(50.8%)	172,800	(52.9%)	182,607	(51.1%)	154,064	(48.3%)
5–14 years old	413,439	(41.2%)	123,920	(37.9%)	149,336	(41.8%)	140,183	(44.0%)
**Economic region (n, %)**								
Northeast	115,570	(11.5%)	38,762	(11.9%)	43,132	(12.1%)	33,676	(10.6%)
East	618,017	(61.6%)	202,141	(61.9%)	225,812	(63.2%)	190,064	(59.6%)
Central	106,459	(10.6%)	37,065	(11.3%)	35,981	(10.1%)	33,413	(10.5%)
West	162,641	(16.2%)	48,649	(14.9%)	52,441	(14.7%)	61,551	(19.3%)
**Type of health insurance or health care plan (n, %)**								
URBMI	178,917	(17.8%)	49,019	(15.0%)	64,993	(18.2%)	64,905	(20.4%)
NRCMS	5274	(0.5%)	1501	(0.5%)	1978	(0.6%)	1795	(0.6%)
CHI	472	(0.0%)	193	(0.1%)	146	(0.0%)	133	(0.0%)
WHC	6458	(0.6%)	2397	(0.7%)	2298	(0.6%)	1763	(0.6%)
PHC	141,413	(14.1%)	40,030	(12.3%)	46,172	(12.9%)	55,211	(17.3%)
OOP	660,292	(65.9%)	229,749	(70.3%)	238,004	(66.6%)	192,539	(60.4%)
Others	9861	(1.0%)	3728	(1.1%)	3775	(1.1%)	2358	(0.7%)
**Type of the consultation (n, %)**								
General consultation	337,423	(33.7%)	109,376	(33.5%)	119,134	(33.3%)	108,913	(34.2%)
Expert consultation	228,325	(22.8%)	80,719	(24.7%)	83,961	(23.5%)	63,645	(20.0%)
General consultation in ED	367,579	(36.7%)	115,947	(35.5%)	128,739	(36.0%)	122,893	(38.6%)
Expert consultation in ED	69,360	(6.9%)	20,575	(6.3%)	25,532	(7.1%)	23,253	(7.3%)
**Classification of AURI (n, %)**								
Acute nasopharyngitis	1094	(0.1%)	268	(0.1%)	452	(0.1%)	374	(0.1%)
Acute sinusitis	3867	(0.4%)	1213	(0.4%)	1262	(0.4%)	1392	(0.4%)
Acute pharyngitis	57,775	(5.8%)	18,331	(5.6%)	19,875	(5.5%)	19,569	(6.1%)
Acute tonsillitis	137,128	(13.7%)	40,537	(12.4%)	48,642	(13.6%)	47,949	(15.0%)
Acute laryngitis, tracheitis & epiglottitis ^a^	70,919	(7.1%)	22,489	(6.9%)	25,707	(7.2%)	22,723	(7.1%)
Unspecified AURI	731,904	(73.0%)	243,779	(74.6%)	261,428	(73.2%)	226,697	(71.1%)

*URBMI* Urban Resident Basic Medical Insurance, *NRCMS* New Rural Cooperative Medical System, *CHI* Commercial Health Insurance, *WHC* Welfare Health Care, *PHC* Preferential Health Care, *OOP* Out-of-pocket Payment, *ED* Emergency department, *AURI* Acute Upper Respiratory Tract Infections

^a^This group comprised the patients with the diagnosis of “acute laryngitis and tracheitis(J04)” and “acute obstructive laryngitis and epiglottitis (J05)”

### Quantity of prescribed medications

A total of 2774 classes of medications were prescribed 2,682,118 times among 938,687 out of 1,002,687 visits. These medications were classified into 146 subcategories of 27 major categories according to the China Pharmacopeia (2015 edition) [10]. The average number of drugs prescribed per visit was 2.8. The most typical situation was prescribing two classes of medications (27.3%) per visit, followed by three (23.9%) and one (21.7%).

**Fig. 1:**
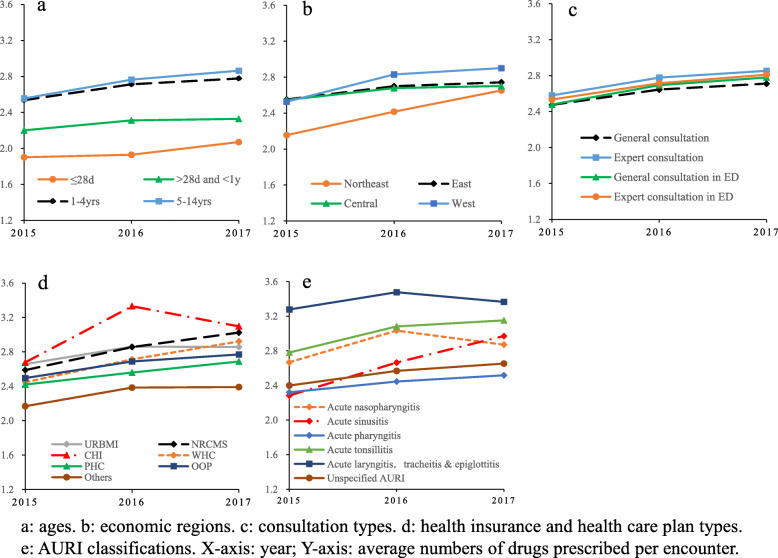
Comparison of average numbers of drugs prescribed per encounter among subgroups in 2015–2017

The average number of medications prescribed per visit appeared upward trends in all subgroups from 2015 to 2017 (Fig. 1). The younger the child was, the fewer the quantity of medication was prescribed, where 48.5% of newborns were prescribed only one medication per visit. The children in the western region received the highest supply of drugs per visit, while those in northeastern hospitals were prescribed with the lowest. CHI and NRCMS beneficiaries were prescribed the largest number of drugs with more than three per encounter, while PHC enrollees were provided the least of 2.71. In cases diagnosed with acute laryngitis, tracheitis & epiglottitis, most medications (an average of 3.3) were prescribed. By comparison, acute sinusitis cases were offered the least medication preparations. The overall differences on the three-year average among subgroups of different ages, regions, health insurances, consultation types, and AURI classifications were all significant (*p* < 0.0001) by Kruskal-Wallis nonparametric test. Further pairwise comparisons showed there was no significant difference (*p* = 0.9597) in the number of medicines prescribed by experts, whether in the emergency department or not. There were also no significant differences between pairs of CHI and NRCMS subgroups (*p* = 0.5029), URBMI and WHC subgroups (*p* = 0.9123), as well as acute nasopharyngitis and acute tonsillitis subgroups (*p* = 0.1106). Apart from those, significant differences were tested in any pair of means (see Additional file 1).

### Categories of prescribed medications

**Fig. 2:**
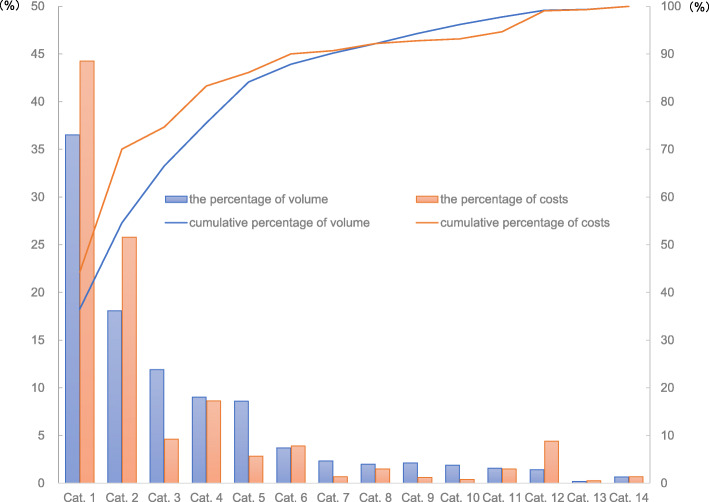
The percentages and cumulative percentages of prescription frequencies and costs of drugs. Cat. 1 = Chinese traditional patent medicine, Cat. 2 = Antibiotic anti-infective drugs, Cat. 3 = Antipyretic-analgesics drugs, Cat. 4 = Respiratory system drugs, Cat. 5 = Water, electrolyte & acid-base balance regulating drugs, Cat. 6 = Non-antibiotic anti-infective drugs#, Cat. 7 = Antiallergic drugs, Cat. 8 = Digestive system drugs, Cat. 9 = Vitamins & minerals, Cat. 10 = Hormones & endocrine regulating drugs, Cat. 11 = Specialist drugs^†^, Cat. 12 = Immunoregulation drugs, Cat. 13 = Chinese herbal medicines, Cat. 14 = Others, # Refer to anti-infective drugs other than antibiotics, such as antiviral drugs, antifungal agents, etc., † Refer to medicines especially for specialist diseases, such as Norfloxacin cream, one of the Dermatology medications

The most frequently prescribed medication was Chinese traditional patent medicines (CTPM), one classification of medicine using Chinese herbal medicines as raw materials and processing into a certain dosage form based on a specific formula. The total amount of CTPM reached 36.5% of overall prescriptions, followed by antibiotics (18.1%) (Fig. 2). The percentage of visits during which children were prescribed CTPM rose from 63.6% in 2015 to 71.4% in 2017, while the figures for antibiotics were from 40.4 to 46.2%. Besides, 17.4% of cases were only prescribed CTPM in 3 years. In the northeast region, there were more children (around 30%) only consuming CTPM.

Then we investigated deeper into the influencing factors of the two commonest prescribed drugs, CTPM and antibiotics, by performing a multinomial logistic regression. The outcome variable in the regression model was sorted into four categories: prescribing CTPM only (including Chinese herbal medicine), prescribing antibiotics only, prescribing both two categories, and prescribing neither one (reference group). The explanatory variables included age, areas, insurance types, consultant types, and AURI classifications. The findings showed that elder children had larger probabilities to be provided both CTPM and antibiotics. The impact of age was larger on antibiotics usage than CTPM. Compared to the Central area, the Northeast physicians were most likely to prescribe both CTPM (OR = 1.29) and antibiotics (OR = 1.64), while the East region proved the least probabilities to prescribe CTPM (OR = 0.69). The Central area itself was the least possible one to prescribe antibiotics. In comparison to OOP patients, the WHC (OR = 0.94 and 0.52) and NCMS (OR = 0.94 and 0.69) beneficiaries were less likely to be provided both CPTM and antibiotics, while other health insurance enrollees were provided more often. Experts were more inclined to prescribe CTPM than general physicians, whether in the emergency department or not. And the experts in emergency department had the least probabilities to prescribe antibiotics (OR = 0.56). In terms of AURI classifications, children diagnosed with acute pharyngitis were provided CTPM more often (OR = 1.2, taking unspecified AURI as reference) and those diagnosed with acute tonsillitis were offered antibiotics more often (OR = 2.6). Complete results of the regression analysis could be found in Additional file 1.

### Prescription diversity

**Fig. 3:**
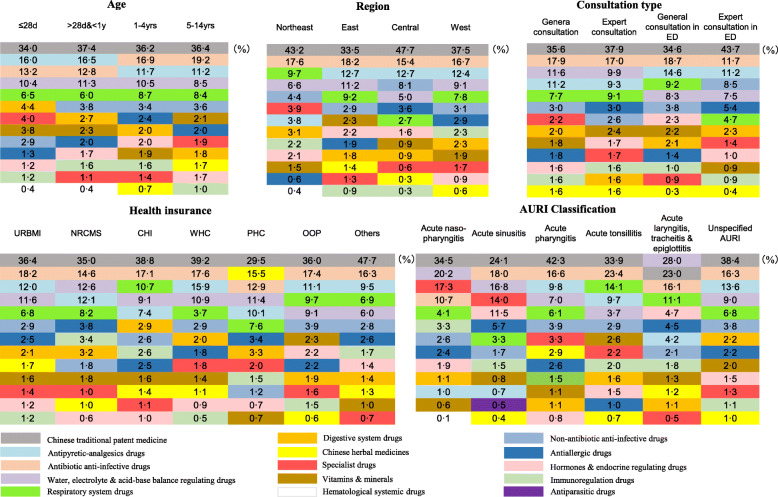
Prescription Diversity among subgroups. The colored squares represent different major categories of drugs; the number in the square shows the percentage of the major category of drugs prescribed in its subgroup

As illustrated in Fig. 3, we used one color to label the same major category of drugs and noted the share of its prescription volume as well. The colored squares were sorted by percentages from top to bottom.

Notably, the use of CTPM to treat AURI was widespread among children in China, regardless of the age, region, insurance type, and consultation type. It ranked first in almost every subgroup. The top five major categories remained stable in each group. Still, disparities existed at the same time. For example, the antipyretic-analgesics medicines were used more often in children under one-year-old than elders. They were prescribed less frequently in the northeast region than in other areas, and they played a more significant role in the emergency departments.

Some subgroups showed their own typical features. For instance, the usage of traditional Chinese medicinal materials in all children groups was relatively small (between 0 and 3%) except for PHC beneficiaries, which was up to 15.5%. In another example, we could find that non-antibiotic anti-infective drugs prescribed by expert physicians in the emergency department were significantly higher than other consultant types. Among different AURI classification groups, the disparity of prescription was prominent.

Five pairs of CTPM and conventional medicines of high frequencies which had similar effects mainly in alleviating symptoms of AURI were chosen to be compared. The conventional medicines referred to those chemosynthetic or single-molecular drugs that were distinguished with Chinese traditional medications. In three out of five pairs, the prescription frequencies of CTPM were 2.1–8.6 times of those of conventional medicines. While in the other two pairs, however, the volumes of conventional medicines were a litter higher than Chinese patent medicines. And the median drug costs per encounter for Chinese patent drugs were higher than those for conventional medicines in all selected pairs (Table 2).

**Table 2:** Comparisons of volume and expenditures between CPTM and conventional medicines with similar effects

	Major Category	Subcategory	Frequency of prescription	Median drug expenditure per visit (USD)
1	Chinese traditional patent medicines	Chinese heat-clearing formula	340,945	$5.79
	Antipyretic-analgesics drugs	Analgesic-antipyretic drugs	49,716	$5.26
2	Chinese traditional patent medicines	Chinese cough-suppressing panting-calming formula	158,845	$4.66
	Respiratory system drugs	Antitussives	49,848	$3.50
3	Chinese traditional patent medicines	Chinese nasal congestion relieving formula	149,133	$9.86
	Specialist drugs	Otorhinolaryngology drugs	17,419	$7.29
4	Chinese traditional patent medicines	Chinese expectorant formula	84,742	$3.88
	Respiratory system drugs	Expectorants	107,254	$3.66
5	Chinese traditional patent medicines	Chinese traditional digestive formula	9160	$6.80
	Digestive system drugs	Stomachic and digestion aid medicine	10,826	$5.59

### Costs of outpatient care for pediatric patients with AURI

**Fig. 4:**
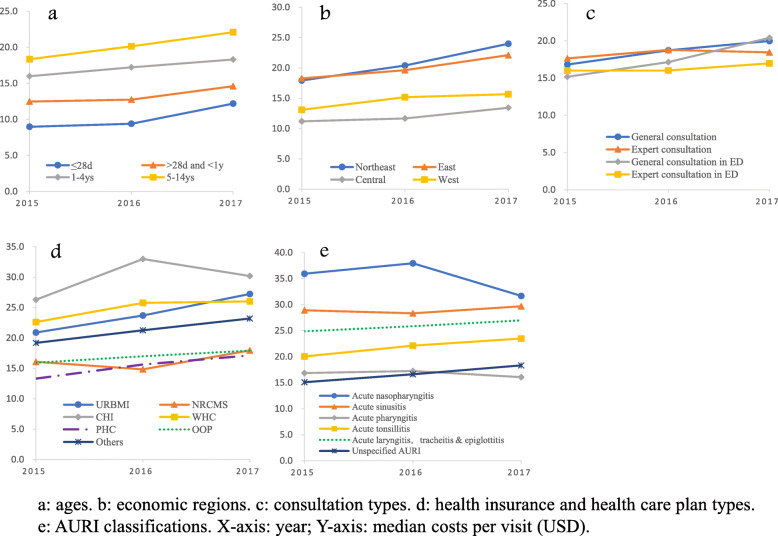
Comparison of median costs per visit among subgroups in 2015–2017

On a per-visit basis, the median cost was 17.91 USD with an average annual growth rate of 5.5% between 2015 and 2017 (Fig. 4). Only two subgroups (acute nasopharyngitis and acute pharyngitis) showed a downward trend in the cost per visit when compared to the year 2017 with 2015. Specifically, the expense on elder children aged 5–14 years (20.22 USD) is nearly twice as much as the expenses of neonates (10.31 USD) and around 1.5 times of that of children younger than one (13.13 USD). Compared with the cases in central (14.79 USD) and west regions (12.01 USD), the expenses of those in the east (19.89 USD) and northeast region (20.39 USD) are much higher. CHI participants (29.08 USD) experienced the highest cost, while the PHC beneficiaries spent the least (15.49 USD). In terms of different classification of AURI, acute nasopharyngitis appeared to be the costliest type of disease (35.16 USD), while unspecified AURI showed the least spending (16.63 USD) which is less than the half of the former. The overall discrepancies of the cost per visit were significant among subgroups of different ages, regions, insurance types, and AURI classifications (*P* < 0.0001 in all Kruskal-Wallis nonparametric tests). The pairwise differences were also statistically significant between any two subgroups except for two pairs of expert and non-expert general consultations (*p* = 0.5513), as well as URBMI and WHS beneficiaries (*p* = 0.6424) (see Additional file 1).

The costly cases that spent more than 77.61 USD (three times of mean costs) took up 4.0% of total consultations, with the highest cost of 146.99 USD. Subgroups covered by CHI and diagnosed with acute sinusitis produced the highest proportions of expensive cases, which was 13.1 and 10.3%, respectively.

The median drug cost per visit was 13.84 USD. The five most costly drugs in one prescription were immunoregulation drugs (13.46 USD), antibiotics (6.05 USD), Chinese traditional patent medicines (5.87 USD), non-antibiotic anti-infective drugs (4.82 USD), and specialty medicines (4.80 USD). In contrast with CTPM, the median spending of Chinese herbal medicine per prescription was only 0.55 USD, being less than 10% of spending on CTPM.

## Discussion

In our results, the average number of medications prescribed per encounter was similar to some studies [21, 22]. It is noteworthy that CTPM stood out of all kinds of drugs, ranking the first in total prescription volume, covering more than two-thirds of the visits. And there was nearly 20% of cases only prescribed CTPM. It suggests a distinct feature of prescriptions in China, compared with results in foreign studies [21, 23–30]. For example, the most commonly prescribed medicines for AURI children in a Indian study were respiratory medicines (47.0%), antimicrobials (30.7%), and analgesic-antipyretics (18.8%) [21], which in our study were only 9.0, 18.1, and 11.9%, respectively, all ranking below CTPM. Moreover, it showed that there was a notable preference for CTPM over conventional medicines with similar effects in alleviating symptoms of AURI, as shown in the comparison analysis (Table 2). However, the drug costs spending on CPTM per visit was not that economical, which is almost the same with antibiotics and higher than non-antibiotic anti-infective drugs as well as other specialty medicines. It is worth noting that, by contrast, the expenditure for Chinese traditional herbal materials is exceedingly low at 0.55 USD, being less than 10% of the CTPM cost. Another issue should be raised in terms of combination use of drugs. Our data showed there were a small proportion of children who were provided more than 5 classes of drugs per visit (10% in 2015 and 15% in 2017). In these cases, the risk of drug-drug interactions between remedies may largely increase. Irrational combination of drugs should be avoided. Because CPTM and other drugs may contain several ingredients with synergetic or antagonistic effects that should be avoided for young children. More clinical studies and economic evaluation concerning the efficacy, safety, and cost-effectiveness of the CTPM drugs are needed.

The finding also showed the overall percentage of child outpatients prescribed antibiotics was 43.8%, which was moderate compared with some existing studies. It was reported to be 58.7% in Korea, 68.1% in India, 66.4% in Japan, 18.9% in Germany, and 46–48% in the United States [24–30]. Compared with previous studies in China, however, vast discrepancies existed. In a systematic review and a meta-analysis [3, 31], the percentage of antibiotic prescriptions was both reported to be more than 80%. In other regional research, it was reported that the rate of antibiotics prescribed for children with AURI was 68% in township hospitals in Guangxi Province, 5.9% in a district hospital in Beijing, and 50% in village health clinics [9, 32, 33]. The possible reason underlying the large discrepancies may be that the previous studies were limited to the sample size and the specific studied facilitate or region.

It was also reported in the previous study that there was a significant reduction of antibiotic consumption after an action plan in China until 2014 [4]. However, in our study, the utilization of antibiotics related to AURI among children showed the opposite tendency between 2015 and 2017. The controversial results may indicate the uncertain effect of such efforts in the case of outpatient care for AURI.

In terms of the costs, the total outpatient care expenditure per encounter of uncomplicated AURI patients remained at a moderate level. But the share of out-of-pocket payment was still dominant though slightly decreased over time. Despite the tremendous progress achieved in promoting universal healthcare in the country, it indicated that the financial support for children’s outpatient care was still not enough. Meanwhile, disparities could be seen in different subgroups, the health insurance type of pediatric patients had an impact on prescriptions and costs. The cost disparities were likely resulted from various types and quantities of services provided to patients based on the physicians’ decisions, considering financial situations of the patients and reimbursement policies of their health plans. More robust payment strategies such as prospective outpatient case-based payment should be designed, controlling the patients’ financial risk by determining a relatively fixed payment amount.

This study mobilized detailed information from patient-level medical records in Chinese hospitals nationwide to describe the current situation of the prescribing pattern and spending. There are several limitations. First, the data source was only from hospitals. However, self-prescribing is still common in many families and they have access for antibiotics and other drugs over the counter outside hospitals, though it is much rarely happened to children compared to adults. These situations were not able to be assessed in the present study. Meanwhile, the information on the real consumption of drugs among children before and after they left the hospitals was not obtained. Therefore, the results of the study were not equal to the actual utilization of medications.

Second, though the selected hospitals are distributed in 31 provinces in mainland China that covered a substantial population and regions, they were not selected randomly. We mainly focus on tertiary public hospitals and did not include facilitates of other degrees. Although consultants happened in tertiary hospitals (17.3 billion) accounted for 57.3% of total outpatient visits in 2017, it could not be representative for hospitals of other levels that may have different prescribing patterns and behavior characteristics. We were also not able to discuss primary health care in the present work. They provided another 310 million pediatric outpatient consultants while hospitals undertook 550 million in 2017 [1]. Third, big data may superiorly overcome some challenges in regional studies and can significantly increase the statistical power of population-based studies. But it still cannot entirely represent the whole situation. Finally, there are other critical factors such as regulatory policies influencing prescription practices and costs that were not considered in our analysis. A subsequent study with factor analyses could help better understand the issues.

## Conclusions

The high volume of CPTM usage is the typical feature in outpatient care of AURI pediatric patients in China. The rational and cost-effective use of CPTM and antibiotics still faces challenges. The reimbursement for child AURI cases needs to be enhanced.

## Supplementary Information

**Additional file 1.**

## Data Availability

The datasets used and/or analyzed during the current study are available from the corresponding author on reasonable request.

## Abbreviations

AURI: Acute upper respiratory tract infection
NLP: Natural language processing
CPTM: Chinese traditional patent medicines
URBMI: Urban Resident Basic Medical Insurance
NRCMS: New Rural Cooperative Medical System
CHI: Commercial Health Insurance
WHC: Welfare Health Care
PHC: Preferential Health Care
OOP: Out-of-pocket Payment
ED: Emergency department
EC: Expert consultation
GC: General consultation
EDEC: Expert consultation in the emergency department
EDGC: General consultation in the emergency department

## References

[1] China National health commission (2018). Chinese statistical yearbook of health care 2018.

[2] Wang Y-Y, Du P, Huang F, Li D-J, Gu J, Shen F-M (2016). Antimicrobial prescribing patterns in a large tertiary hospital in Shanghai, China. Int J Antimicrob Agents.

[3] Li J, Song X, Yang T, Chen Y, Gong Y, Yin X (2016). A systematic review of antibiotic prescription associated with upper respiratory tract infections in China. Medicine (Baltimore).

[4] Bao L, Peng R, Wang Y, Ma R, Ren X, Meng W (2015). Significant reduction of antibiotic consumption and patients’ costs after an action plan in China, 2010–2014. PLoS One.

[5] Hui L, Li X-S, Zeng X-J, Dai Y-H, Foy HM (1997). Patterns and determinants of use of antibiotics for acute respiratory tract infection in children in China. Pediatr Infect Dis J.

[6] Liu XX, Li Y, Zhu Y (2017). Seasonal pattern of influenza activity in a subtropical city, China, 2010-2015. Sci Rep.

[7] Yang J, Jit M, Leung KS (2015). The economic burden of influenza-associated outpatient visits and hospitalizations in China: a retrospective survey. Infect Dis Poverty.

[8] Liang X, Xia T, Zhang X, Jin C (2014). Governance structure reform and antibiotics prescription in community health centres in Shenzhen, China. Fam Pract.

[9] Wang J, Wang P, Wang X, Zheng Y, Xiao Y (2014). Use and prescription of antibiotics in primary health care settings in China. JAMA Intern Med.

[10] Mao W, Vu H, Xie Z, Chen W, Tang S (2015). Systematic review on irrational use of medicines in China and Vietnam. PLoS One.

[11] Wu Y, Yang C, Xi H, Zhang Y, Zhou Z, Hu Y (2016). Prescription of antibacterial agents for acute upper respiratory tract infections in Beijing, 2010-2012. Eur J Clin Pharmacol.

[12] Yin X, Song F, Gong Y (2013). A systematic review of antibiotic utilization in China. J Antimicrob Chemother.

[13] Li X, Lu J, Hu S (2017). The primary health-care system in China. Lancet.

[14] Li HM, Chen YC, Gao HX (2018). Effectiveness evaluation of quota payment for specific diseases under global budget: a typical provider payment system reform in rural China. BMC Health Serv Res.

[15] Zhang A, Nikoloski Z, Mossialos E (2017). Does health insurance reduce out-of-pocket expenditure? Heterogeneity among China's middle-aged and elderly. Soc Sci Med.

[16] International Statistical Classification of Diseases and Related Health Problems-10th revision (Chinese edition 2012). China National Health Commission; http://www.nhc.gov.cn/mohwsbwstjxxzx/s8553/201202/54034.shtml. Accessed 5 Nov 2018.

[17] Weng WH, Wagholikar KB, McCray AT, Szolovits P, Chueh HC (2017). Medical subdomain classification of clinical notes using a machine learning-based natural language processing approach. BMC Med Inform Decis Mak.

[18] Chinese Pharmacopoeia Commission: Chinese Pharmacopoeia (2015 edn.). http://eng.sfda.gov.cn/WS03/CL0757/122060.html. Accessed 5 Dec 2018.

[19] National Bureau of Statistics of China: The partition of China’s economic regions. http://www.stats.gov.cn/ztjc/zthd/sjtjr/dejtjkfr/tjkp/201106/t2011061371947.htm. Accessed 1 Dec 2018.

[20] Lin W, Liu GG, Chen G (2009). The urban resident basic medical insurance: a landmark reform towards universal coverage in China. Health Econ.

[21] Das B, Sarkar C, Majumder AG. Medication use for pediatric upper respiratory tract infections. Fundamental & clinical pharmacology. 2006 Aug;20(4):385-90.. 10.1111/j.1472-8206.2006.00414.x 16867023

[22] Pandey AA, Thakre SB, Bhatkule PR (2010). Prescription analysis of pediatric outpatient practice in Nagpur city. Indian J Community Med.

[23] Maltezou HC, Dedoukou X, Asimaki H (2017). Consumption of antibiotics by children in Greece: a cross-sectional study. Int J Pediatr Adolesc Med.

[24] Shin SM, Shin J-Y, Kim MH, Lee SH, Choi S, Park B-J (2015). Prevalence of antibiotic use for pediatric acute upper respiratory tract infections in Korea. J Korean Med Sci.

[25] Majhi B, Panda A, Barma SK (2017). Antibiotic prescribing pattern in paediatrics outpatient in a tertiary care hospital. J Evid Based Med.

[26] Yoshida S, Takeuchi M, Kawakami K (2017). Prescription of antibiotics to pre-school children from 2005 to 2014 in Japan: a retrospective claims database study. J Public Health (Oxf).

[27] Schindler C, Krappweis J, Morgenstern I, Kirch W (2003). Prescriptions of systemic antibiotics for children in Germany aged between 0 and 6 years. Pharmacoepidemiol Drug Saf.

[28] Nyquist A-C, Gonzales R, Steiner JF, Sande MA (1998). Antibiotic prescribing for children with colds, upper respiratory tract infections, and bronchitis. JAMA..

[29] Agiro A, Gautam S, Wall E, Hackell J, Helm M, Barron J (2018). Variation in outpatient antibiotic dispensing for respiratory infections in children by clinician specialty and treatment setting. Pediatr Infect Dis J.

[30] Fleming-Dutra KE, Hersh AL, Shapiro DJ (2016). Prevalence of inappropriate antibiotic prescriptions among US ambulatory care visits, 2010-2011. JAMA..

[31] Li WM, Lu YL, Chen MY, Yin G, Zeng XY (2017). Usage of antibiotics among children with upper respiratory tract infections in China before and after the new health care reforms: Meta Analysis. Chinese Pharmaceut J.

[32] Zhang Z, Hu Y, Zou G, Lin M, Zeng J, Deng S (2017). Antibiotic prescribing for upper respiratory infections among children in rural China: a cross-sectional study of outpatient prescriptions. Glob Health Action.

[33] Dong L, Yan H, Wang D (2008). Antibiotic prescribing patterns in village health clinics across 10 provinces of Western China. J Antimicrob Chemother.

