# Supplementary material for: Characteristics of prescriptions and costs for acute upper respiratory tract infections in Chinese outpatient pediatric patients: a nationwide cross-sectional study
Source: BMC Complement Med Ther. 2020 Nov 16;20:346. doi: 10.1186/s12906-020-03141-w (PMC7667745; doi:10.1186/s12906-020-03141-w)
Supplement: Supplementary file 1 — Additional file 1. [file 12906_2020_3141_MOESM1_ESM.docx]

**Appendix**

**1 Brief introduction of health insurance and payment system of China**

China has implemented basic social health insurance system, including Urban Employee Basic Medical Insurance (UEBMI, established in 1998) for employment population in the formal sector in cities, New Rural Cooperative Medical System (NRCMS, established in 2003), for rural residents, and Urban Resident Basic Medical Insurance (URBMI, established in 2007) for unemployed urban residents according to household registration status. They have covered more than 97% population by the year of 2015 ^[[1]](#footnote-1)^. URBMI and NCMS are applicable to urban and rural children, respectively. These three schemes are the only health insurance for most citizens. There is also a social health plan for serious diseases. Apart from these, there are various supplementary health insurance or health plans, which may benefit specific groups of people, such as specific occupations. And different provinces or cities may have their own plans. Commercial health insurance was less common in the country. Only Urban Employee Basic Medical Insurance (UEBMI) is comprehensive for both outpatient and inpatient care, whereas the other two plans, New Rural Cooperative Medical System (NRCMS) and Urban Resident Basic Medical Insurance (URBMI) primarily cover inpatient care and selected outpatient services ^[[2]](#footnote-2)^. Fee-for-service payment method is still the main approach adopted in all types of health care in China. While diversiform provider payment reforms have been widely implemented for inpatient care at different levels across the country, such as case-based payment, per diem payment, etc., the outpatient care in hospitals are dominantly reimbursed on a traditional fee-for-service basis with less reform. Moreover, in many cases, patients need to pay for the bill directly to the hospital at the first time and seek for reimbursement afterwards.

**2 Supplementary information of standardization with NLP**

***Diagnosis information standardization:***

*Step 1:*Within the 410 thousand original entries of diagnoses , 16.2 thousand invalid diagnoses and nonsense characters were removed.

Step 2:According to the 10th revision of the International Statistical Classification of Diseases and Related Health Problems (ICD-10, Chinese 2011 edition),19 Chinese keywords for all types of AURI diagnoses were extracted from 39 names of disease classification in the J00-J06 code section.

Step 3: The cleaned original diagnosis entries in step 1 were distinguished through regular expression string matching with keywords^[[3]](#footnote-3)^. They were automatically classified and standardized once matched.

Step 4: There were 7498 kinds of descriptions recognized and encoded into seven main AURI classifications. Only after diagnoses standardization procedure could AURI patients be identified.

***Prescription standardization:*** The original names of medications on prescriptions went through the same procedure as diagnoses did, all prescriptions were matched with keywords extracted from China Pharmacopeia (2015 edition). Considering the complexity of medication name processing using NLP, we conducted a second round of manual review by pharmacists and a third round of validation by senior pharmacists after initial standardization after the automatic matching procedure. Finally, the medications prescribed to AURI patients were standardized into 2774 classes.

**3 Supplementary results of Student-Newman-Keuls tests**

Tables 1-5 present p values in Student-Newman-Keuls tests for pairwise comparisons of average costs per visit among subgroups by age, regions, consultation types, health insurances, and AURI classifications.

Table 1: p values in Student-Newman-Keuls tests for pairwise comparisons of average costs per visit among subgroups by **ages**

| **Subgroup** | **≤28d** | **>28d and <1y** | **1-4yrs** | **5-14yrs** |
| --- | --- | --- | --- | --- |
| **≤28d** | — | <.0001 | <.0001 | <.0001 |
| **>28d and <1y** | <.0001 | — | <.0001 | <.0001 |
| **1-4yrs** | <.0001 | <.0001 | — | <.0001 |
| **5-14yrs** | <.0001 | <.0001 | <.0001 | — |

Table 2: p values in Student-Newman-Keuls tests for pairwise comparisons of average costs per visit among subgroups by **regions**

| **Subgroup** | **Northeast** | **East** | **West** | **Central** |
| --- | --- | --- | --- | --- |
| **Northeast** | — | <.0001 | <.0001 | <.0001 |
| **East** | <.0001 | — | <.0001 | <.0001 |
| **West** | <.0001 | <.0001 | — | <.0001 |
| **Central** | <.0001 | <.0001 | <.0001 | — |

Table 3: p values in Student-Newman-Keuls tests for pairwise comparisons of average costs per visit among subgroups by **consultation types**

| **Subgroup** | **EDGC** | **EDEC** | **GC** | **EC** |
| --- | --- | --- | --- | --- |
| **EDGC** | — | 0.0003 | <.0001 | <.0001 |
| **EDEC** | 0.0003 | — | <.0001 | <.0001 |
| **GC** | <.0001 | <.0001 | — | 0.5513 |
| **EC** | <.0001 | <.0001 | 0.5513 | — |

Table 4: p values in Student-Newman-Keuls tests for pairwise comparisons of average costs per visit among subgroups by **health insurance types**

| **Subgroup** | **URBMI** | **WHC** | **Others** | **CMI** | **NRCMS** | **PHC** | **OOP** |
| --- | --- | --- | --- | --- | --- | --- | --- |
| **URBMI** | — | 0.6424 | <.0001 | <.0001 | <.0001 | <.0001 | <.0001 |
| **WHC** | 0.6424 | — | <.0001 | 0.0002 | <.0001 | <.0001 | <.0001 |
| **Others** | <.0001 | <.0001 | — | <.0001 | <.0001 | <.0001 | <.0001 |
| **CMI** | <.0001 | 0.0002 | <.0001 | — | <.0001 | <.0001 | <.0001 |
| **NRCMS** | <.0001 | <.0001 | <.0001 | <.0001 | — | <.0001 | 0.0438 |
| **PHC** | <.0001 | <.0001 | <.0001 | <.0001 | <.0001 | — | <.0001 |
| **OOP** | <.0001 | <.0001 | <.0001 | <.0001 | 0.0438 | <.0001 | — |

Table 5: p values in Student-Newman-Keuls tests for pairwise comparisons of average costs per visit among subgroups by **AURI classifications**

| **Subgroup** | **J00** | **J01** | **J02** | **J03** | **J04-05** | **J06** |
| --- | --- | --- | --- | --- | --- | --- |
| **J00** | — | <.0001 | <.0001 | <.0001 | <.0001 | <.0001 |
| **J01** | <.0001 | — | <.0001 | <.0001 | <.0001 | <.0001 |
| **J02** | <.0001 | <.0001 | — | <.0001 | <.0001 | <.0001 |
| **J03** | <.0001 | <.0001 | <.0001 | — | <.0001 | <.0001 |
| **J04** | <.0001 | <.0001 | <.0001 | <.0001 | — | <.0001 |
| **J05** | <.0001 | <.0001 | <.0001 | <.0001 | <.0001 | — |
| **J06** | <.0001 | <.0001 | <.0001 | <.0001 | <.0001 | <.0001 |

Tables 6-10 present p values in Student-Newman-Keuls tests for pairwise comparisons of average numbers of drugs prescribed per visit among subgroups by age, regions, consultation types, health insurances, and AURI classifications.

Table 6: p values in Student-Newman-Keuls tests for pairwise comparisons of average numbers of drugs prescribed per visit among subgroups by **ages**

| **Subgroup** | **≤28d** | **>28d and <1y** | **1-4yrs** | **5-14yrs** |
| --- | --- | --- | --- | --- |
| **≤28d** | — | <.0001 | <.0001 | <.0001 |
| **>28d and <1y** | <.0001 | — | <.0001 | <.0001 |
| **1-4yrs** | <.0001 | <.0001 | — | <.0001 |
| **5-14yrs** | <.0001 | <.0001 | <.0001 | — |

Table 7: p values in Student-Newman-Keuls tests for pairwise comparisons of average numbers of drugs prescribed per visit among subgroups by **regions**

| **Subgroup** | **Northeast** | **East** | **West** | **Central** |
| --- | --- | --- | --- | --- |
| **Northeast** | — | <.0001 | <.0001 | <.0001 |
| **East** | <.0001 | — | <.0001 | <.0001 |
| **West** | <.0001 | <.0001 | — | <.0001 |
| **Central** | <.0001 | <.0001 | <.0001 | — |

Table 8: p values in Student-Newman-Keuls tests for pairwise comparisons of average numbers of drugs prescribed per visit among subgroups by **consultation types**

| **Subgroup** | **EDGC** | **EDEC** | **GC** | **EC** |
| --- | --- | --- | --- | --- |
| **EDGC** | — | <.0001 | <.0001 | <.0001 |
| **EDEC** | <.0001 | — | <.0001 | 0.9597 |
| **GC** | <.0001 | <.0001 | — | <.0001 |
| **EC** | <.0001 | 0.9597 | <.0001 | — |

Table 9: p values in Student-Newman-Keuls tests for pairwise comparisons of average numbers of drugs prescribed per visit among subgroups by **health insurance types**

| **Subgroup** | **URBMI** | **WHC** | **Others** | **CMI** | **NRCMS** | **PHC** | **OOP** |
| --- | --- | --- | --- | --- | --- | --- | --- |
| **URBMI** | — | 0.9123 | <.0001 | <.0001 | <.0001 | <.0001 | <.0001 |
| **WHC** | 0.9123 | — | <.0001 | 0.0007 | <.0001 | <.0001 | <.0001 |
| **Others** | <.0001 | <.0001 | — | <.0001 | <.0001 | <.0001 | <.0001 |
| **CMI** | <.0001 | 0.0007 | <.0001 | — | 0.5029 | <.0001 | <.0001 |
| **NRCMS** | <.0001 | <.0001 | <.0001 | 0.5029 | — | <.0001 | <.0001 |
| **PHC** | <.0001 | <.0001 | <.0001 | <.0001 | <.0001 | — | <.0001 |
| **OOP** | <.0001 | <.0001 | <.0001 | <.0001 | <.0001 | <.0001 | — |

Table 10: p values in Student-Newman-Keuls tests for pairwise comparisons of average numbers of drugs prescribed per visit among subgroups by **AURI classifications**

| **Subgroup** | **J00** | **J01** | **J02** | **J03** | **J04-05** | **J06** |
| --- | --- | --- | --- | --- | --- | --- |
| **J00** | — | <.0001 | <.0001 | 0.1106 | <.0001 | <.0001 |
| **J01** | <.0001 | — | <.0001 | <.0001 | <.0001 | 0.0019 |
| **J02** | <.0001 | <.0001 | — | <.0001 | <.0001 | <.0001 |
| **J03** | 0.1106 | <.0001 | <.0001 | — | <.0001 | <.0001 |
| **J04** | <.0001 | <.0001 | <.0001 | <.0001 | — | <.0001 |
| **J05** | <.0001 | 0.0019 | <.0001 | <.0001 | <.0001 | — |
| **J06** | <.0001 | <.0001 | <.0001 | 0.1106 | <.0001 | <.0001 |

**4 The results of multinomial logistic regresson.**

**Table 11:** Testing of Model

| Test | chi-square | Degree of Freedom | Pr > ChiSq |
| --- | --- | --- | --- |
| Likelihood Ratio | 80514.2852 | 60 | <.0001 |
| Score | 78425.4256 | 60 | <.0001 |
| Wald | 73139.8362 | 60 | <.0001 |

**Table 12 : Estimates of varaibles**

| Parameter | | Y* | Estimate | Standard Error | Wald Chi-Square | Pr > Chisq |
| --- | --- | --- | --- | --- | --- | --- |
| Intercept |  | 1 | 0.8143 | 0.032 | 646.4874 | <.0001 |
| Intercept |  | 2 | -0.5809 | 0.0415 | 196.0724 | <.0001 |
| Intercept |  | 3 | 0.1248 | 0.037 | 11.3776 | 0.0007 |
| AGE | 1-4 years old | 1 | 0.2569 | 0.0169 | 229.9881 | <.0001 |
| AGE | 1-4 years old | 2 | 0.3217 | 0.0234 | 188.2593 | <.0001 |
| AGE | 1-4 years old | 3 | 0.5503 | 0.0232 | 560.8312 | <.0001 |
| AGE | >28 days and <1year | 1 | 0.0609 | 0.0179 | 11.6048 | 0.0007 |
| AGE | >28 days and <2year | 2 | -0.2675 | 0.0249 | 115.8185 | <.0001 |
| AGE | >28 days and <3year | 3 | -0.3075 | 0.0243 | 159.6688 | <.0001 |
| AGE | 5-14 years old | 1 | 0.3517 | 0.0172 | 415.954 | <.0001 |
| AGE | 5-14 years old | 2 | 0.5954 | 0.0237 | 630.627 | <.0001 |
| AGE | 5-14 years old | 3 | 0.8811 | 0.0235 | 1411.0734 | <.0001 |
| AREA | Northeast | 1 | 0.3648 | 0.00983 | 1377.2308 | <.0001 |
| AREA | Northeast | 2 | 0.2498 | 0.0117 | 454.8599 | <.0001 |
| AREA | Northeast | 3 | 0.0537 | 0.0104 | 26.6829 | <.0001 |
| AREA | East | 1 | -0.2626 | 0.006 | 1914.9519 | <.0001 |
| AREA | East | 2 | 0.0839 | 0.00729 | 132.5886 | <.0001 |
| AREA | East | 3 | -0.0541 | 0.00624 | 75.1318 | <.0001 |
| AREA | West | 1 | -0.2129 | 0.00681 | 975.7974 | <.0001 |
| AREA | West | 2 | -0.092 | 0.0083 | 122.9217 | <.0001 |
| AREA | West | 3 | -0.2302 | 0.00714 | 1040.6366 | <.0001 |
| INSURANCE | URBMI | 1 | 0.069 | 0.023 | 9.04 | 0.0026 |
| INSURANCE | URBMI | 2 | 0.1492 | 0.0267 | 31.2973 | <.0001 |
| INSURANCE | URBMI | 3 | 0.2777 | 0.0232 | 143.3954 | <.0001 |
| INSURANCE | WHC | 1 | -0.2332 | 0.0392 | 35.3436 | <.0001 |
| INSURANCE | WHC | 2 | -0.5658 | 0.0486 | 135.5861 | <.0001 |
| INSURANCE | WHC | 3 | -0.6196 | 0.0412 | 226.3853 | <.0001 |
| INSURANCE | Others | 1 | 0.396 | 0.038 | 108.7716 | <.0001 |
| INSURANCE | Others | 2 | 0.188 | 0.046 | 16.6953 | <.0001 |
| INSURANCE | Others | 3 | 0.0603 | 0.0405 | 2.2188 | 0.1363 |
| INSURANCE | CHI | 1 | 0.1659 | 0.1192 | 1.9371 | 0.164 |
| INSURANCE | CHI | 2 | 0.3083 | 0.1363 | 5.1151 | 0.0237 |
| INSURANCE | CHI | 3 | 0.5586 | 0.119 | 22.0281 | <.0001 |
| INSURANCE | NCMS | 1 | -0.229 | 0.0339 | 45.5661 | <.0001 |
| INSURANCE | NCMS | 2 | -0.2769 | 0.0414 | 44.6774 | <.0001 |
| INSURANCE | NCMS | 3 | -0.2516 | 0.0351 | 51.376 | <.0001 |
| INSURANCE | PHC | 1 | 0.00129 | 0.0232 | 0.0031 | 0.9557 |
| INSURANCE | PHC | 2 | 0.1007 | 0.027 | 13.9469 | 0.0002 |
| INSURANCE | PHC | 3 | -0.015 | 0.0235 | 0.4064 | 0.5238 |
| CONSULT | General consultation in ED | 1 | -0.2658 | 0.00589 | 2033.6337 | <.0001 |
| CONSULT | General consultation in ED | 2 | 0.1016 | 0.00736 | 190.5149 | <.0001 |
| CONSULT | General consultation in ED | 3 | 0.0162 | 0.00619 | 6.8349 | 0.0089 |
| CONSULT | Expert consultation in ED | 1 | 0.2012 | 0.011 | 335.9199 | <.0001 |
| CONSULT | Expert consultation in ED | 2 | -0.3783 | 0.0151 | 627.7267 | <.0001 |
| CONSULT | Expert consultation in ED | 3 | -0.224 | 0.0121 | 343.7059 | <.0001 |
| CONSULT | General consultation | 1 | -0.1178 | 0.0061 | 373.3289 | <.0001 |
| CONSULT | General consultation | 2 | 0.0654 | 0.00758 | 74.4726 | <.0001 |
| CONSULT | General consultation | 3 | -0.1002 | 0.00646 | 240.3658 | <.0001 |
| AURI | Acute nasopharyngitis | 1 | -0.3014 | 0.0729 | 17.0845 | <.0001 |
| AURI | Acute nasopharyngitis | 2 | -0.6175 | 0.1075 | 32.9957 | <.0001 |
| AURI | Acute nasopharyngitis | 3 | -0.588 | 0.0853 | 47.5104 | <.0001 |
| AURI | Acute sinusitis | 1 | -0.712 | 0.0425 | 281.1251 | <.0001 |
| AURI | Acute sinusitis | 2 | -0.5176 | 0.0493 | 110.1222 | <.0001 |
| AURI | Acute sinusitis | 3 | -0.8385 | 0.0439 | 365.0452 | <.0001 |
| AURI | Acute pharyngitis | 1 | 0.6393 | 0.0208 | 946.5382 | <.0001 |
| AURI | Acute pharyngitis | 2 | 0.1237 | 0.0277 | 19.9371 | <.0001 |
| AURI | Acute pharyngitis | 3 | 0.2137 | 0.023 | 86.5195 | <.0001 |
| AURI | Acute tonsillitis | 1 | 0.4096 | 0.0194 | 445.5934 | <.0001 |
| AURI | Acute tonsillitis | 2 | 0.9932 | 0.0255 | 1521.6987 | <.0001 |
| AURI | Acute tonsillitis | 3 | 1.1721 | 0.0211 | 3079.4226 | <.0001 |
| AURI | Acute laryngitis, tracheitis & epiglottitis | 1 | -0.4857 | 0.0198 | 602.7401 | <.0001 |
| AURI | Acute laryngitis, tracheitis & epiglottitis | 2 | -0.0171 | 0.0259 | 0.4363 | 0.5089 |
| AURI | Acute laryngitis, tracheitis & epiglottitis | 3 | -0.00474 | 0.0213 | 0.0494 | 0.824 |

*Y=1 represents for prescribing CTPM(including Chinese herbal medicine) only; Y=2 represents for prescribing antibiotics only; Y=3 represents for prescribing both two categories; and Y=4 represents for prescribing neither one (reference group).

**Table 13: Point estimate of all indepent variables**

| Effect | | Y* | Point Estimate | 95% Wald Confidence Limits | |
| --- | --- | --- | --- | --- | --- |
| AGE | 1-4 years old | 1 | 2.525 | 2.22 | 2.872 |
| reference: <28 days | 1-4 years old | 2 | 2.641 | 2.208 | 3.16 |
|  | 1-4 years old | 3 | 5.335 | 4.462 | 6.378 |
|  | >28 days and <1year | 1 | 2.076 | 1.824 | 2.363 |
|  | >28 days and <1year | 2 | 1.465 | 1.223 | 1.755 |
|  | >28 days and <1year | 3 | 2.262 | 1.89 | 2.708 |
|  | 5-14 years old | 1 | 2.777 | 2.441 | 3.159 |
|  | 5-14 years old | 2 | 3.473 | 2.902 | 4.156 |
|  | 5-14 years old | 3 | 7.426 | 6.21 | 8.88 |
| AREA | Northeast | 1 | 1.289 | 1.247 | 1.334 |
| reference: Central | Northeast | 2 | 1.635 | 1.566 | 1.706 |
|  | Northeast | 3 | 0.838 | 0.809 | 0.868 |
|  | East | 1 | 0.688 | 0.67 | 0.707 |
|  | East | 2 | 1.385 | 1.338 | 1.434 |
|  | East | 3 | 0.752 | 0.732 | 0.773 |
|  | West | 1 | 0.724 | 0.704 | 0.744 |
|  | West | 2 | 1.162 | 1.12 | 1.205 |
|  | West | 3 | 0.631 | 0.613 | 0.649 |
| INSURANCE | URBMI | 1 | 1.27 | 1.246 | 1.295 |
| reference: OOP | URBMI | 2 | 1.054 | 1.031 | 1.078 |
|  | URBMI | 3 | 1.334 | 1.308 | 1.36 |
|  | WHC | 1 | 0.939 | 0.87 | 1.013 |
|  | WHC | 2 | 0.516 | 0.468 | 0.568 |
|  | WHC | 3 | 0.544 | 0.501 | 0.59 |
|  | Others | 1 | 1.761 | 1.638 | 1.894 |
|  | Others | 2 | 1.096 | 1.002 | 1.199 |
|  | Others | 3 | 1.073 | 0.991 | 1.162 |
|  | CHI | 1 | 1.399 | 1.066 | 1.836 |
|  | CHI | 2 | 1.236 | 0.906 | 1.686 |
|  | CHI | 3 | 1.766 | 1.347 | 2.317 |
|  | NCMS | 1 | 0.943 | 0.887 | 1.002 |
|  | NCMS | 2 | 0.688 | 0.638 | 0.743 |
|  | NCMS | 3 | 0.786 | 0.737 | 0.837 |
|  | PHC | 1 | 1.187 | 1.163 | 1.212 |
|  | PHC | 2 | 1.004 | 0.98 | 1.029 |
|  | PHC | 3 | 0.995 | 0.974 | 1.017 |
| CONSULTATION TYPE | General consultation in ED | 1 | 0.639 | 0.627 | 0.65 |
| reference: Expert consultation | General consultation in ED | 2 | 0.896 | 0.877 | 0.915 |
|  | General consultation in ED | 3 | 0.747 | 0.733 | 0.761 |
|  | Expert consultation in ED | 1 | 1.019 | 0.988 | 1.051 |
|  | Expert consultation in ED | 2 | 0.555 | 0.532 | 0.578 |
|  | Expert consultation in ED | 3 | 0.587 | 0.568 | 0.608 |
|  | General consultation | 1 | 0.741 | 0.728 | 0.754 |
|  | General consultation | 2 | 0.864 | 0.846 | 0.883 |
|  | General consultation | 3 | 0.665 | 0.653 | 0.677 |
| AURI CLASSIFICATION | Acute nasopharyngitis | 1 | 0.472 | 0.398 | 0.559 |
| reference:Unspecified AURI | Acute nasopharyngitis | 2 | 0.521 | 0.405 | 0.67 |
|  | Acute nasopharyngitis | 3 | 0.531 | 0.435 | 0.648 |
|  | Acute sinusitis | 1 | 0.313 | 0.285 | 0.344 |
|  | Acute sinusitis | 2 | 0.575 | 0.518 | 0.639 |
|  | Acute sinusitis | 3 | 0.413 | 0.376 | 0.455 |
|  | Acute pharyngitis | 1 | 1.208 | 1.172 | 1.245 |
|  | Acute pharyngitis | 2 | 1.092 | 1.053 | 1.133 |
|  | Acute pharyngitis | 3 | 1.183 | 1.146 | 1.221 |
|  | Acute tonsillitis | 1 | 0.96 | 0.937 | 0.983 |
|  | Acute tonsillitis | 2 | 2.606 | 2.541 | 2.672 |
|  | Acute tonsillitis | 3 | 3.085 | 3.015 | 3.157 |
|  | Acute laryngitis, tracheitis & epiglottiti | 1 | 0.392 | 0.383 | 0.402 |
|  | Acute laryngitis, tracheitis & epiglottitis | 2 | 0.949 | 0.924 | 0.975 |
|  | Acute laryngitis, tracheitis & epiglottitis | 3 | 0.951 | 0.929 | 0.973 |

*Y=1 represents for prescribing CTPM(including Chinese herbal medicine) only; Y=2 represents for prescribing antibiotics only; Y=3 represents for prescribing both two categories; and Y=4 represents for prescribing neither one (reference group).

**5 The influence of laboratory test results on physicians’ decisions for antibiotics prescribing**

Among all children who were prescribed antibiotics, 48.6% had taken laboratory tests, 46.9% had taken WBC tests, and 19.4% had taken CRP tests. The children who had taken the WBC test with a positive result accounted for 33.7% of the total amount of children prescribed antibiotics, and it was 13.1% for CRP. This percentage depended on both the rate of laboratory tests taken and the positive rate of the tests. Differences had been shown in subgroups according to categorical variables. Children prescribed antibiotics in subgroups of the northeast region, URBMI, NRCMS, and expert consultation took more lab tests than average. The rates of positive results of the WBC test and CRP test were both high in subgroups of west region (94.6% and 99.3%), NRCMS (94.8% and 95.5%), CHI (94.7% and 96.8%), expert consultant in ED (81.1% and 90.1%) and acute nasopharyngitis (97.9% and 92.2%). NRCMS and CHI were the only two subgroups in which the proportions of children who had taken the WBC test with a positive result and who had taken the CRP test with a positive result were both markedly higher than the average level.

Table 14: The percentages of children taking specific lab tests and the percentages of children with WBC and CRP results among children prescribed antibiotics

|  | % of lab tests taken among children prescribed antibiotics | % of WBC tests taken among children prescribed antibiotics | % of children with positive WBC test result among children prescribed antibiotics | % of CRP tests taken among children prescribed antibiotics | % of children with positive CRP test result among children prescribed antibiotics |
| --- | --- | --- | --- | --- | --- |
| Total | 48.6 | 46.9 | 33.7 | 19.4 | 13.1 |
| Age |  |  |  |  |  |
| ≤28 days | 42.6 | 42.1 | 27.1 | 12.9 | 7.8 |
| >28 days and <1year | 50.0 | 49.1 | 37.0 | 19.7 | 14.2 |
| 1-4 years old | 49.2 | 47.9 | 34.8 | 19.3 | 13.3 |
| 5-14 years old | 47.8 | 45.6 | 32.0 | 19.4 | 12.6 |
| Economic Region |  |  |  |  |  |
| Northeast | 58.5 | 56.4 | 38.6 | 23.9 | 14.4 |
| East | 30.1 | 28.9 | 15.7 | 6.1 | 3.8 |
| Central | 33.4 | 32.4 | 26.2 | 11.7 | 9.5 |
| West | 40.4 | 38.8 | 36.7 | 22.1 | 22.0 |
| Health insurance or healthcare plan |  |  |  |  |  |
| URBMI | 60.6 | 59.1 | 39.0 | 31.8 | 14.4 |
| NRCMS | 56.2 | 54.0 | 51.2 | 39.0 | 37.3 |
| CHI | 40.0 | 37.4 | 26.1 | 12.7 | 8.3 |
| WHC | 53.3 | 50.5 | 47.0 | 4.8 | 2.2 |
| PHC | 53.2 | 47.2 | 44.7 | 36.2 | 35.1 |
| OOP | 48.7 | 39.4 | 37.6 | 11.5 | 8.8 |
| Others | 46.8 | 45.3 | 33.3 | 17.1 | 13.3 |
| Consultation Type |  |  |  |  |  |
| General consultation | 45.0 | 42.9 | 30.1 | 18.0 | 10.6 |
| Expert consultation | 54.6 | 52.3 | 36.4 | 15.4 | 12.7 |
| General consultation in ED | 48.0 | 47.0 | 34.7 | 23.5 | 15.1 |
| Expert consultation in ED | 48.0 | 46.1 | 37.4 | 19.0 | 17.1 |
| AURI Classification |  |  |  |  |  |
| Acute nasopharyngitis | 38.6 | 38.0 | 35.0 | 28.2 | 27.6 |
| Acute sinusitis | 12.8 | 12.0 | 9.3 | 5.1 | 3.8 |
| Acute pharyngitis | 48.1 | 46.1 | 30.6 | 10.8 | 7.6 |
| Acute tonsillitis | 43.0 | 41.6 | 32.9 | 20.3 | 16.0 |
| Acute laryngitis, tracheitis & epiglottitis* | 40.9 | 39.7 | 25.2 | 20.5 | 7.9 |
| Unspecified AURI | 51.8 | 49.9 | 35.4 | 19.6 | 13.2 |
| * This group comprised the patients with the diagnosis of “acute laryngitis and tracheitis(J04)” and “acute obstructive laryngitis and epiglottitis (J05)”.  URBMI=Urban Resident Basic Medical Insurance. NRCMS=New Rural Cooperative Medical System. CHI=Commercial Health Insurance. WHC=Welfare Health Care. PHC=Preferential Health Care. OOP=Out-of-pocket Payment. ED=Emergency department. ARUI=Acute Respiratory Upper Tract Infections. | | | | | |

1. Li L, Fu H. China's health care system reform: Progress and prospects. The International journal of health planning and management. 2017 Jul;32(3):240-53. [↑](#footnote-ref-1)
2. Liu GG, Vortherms SA, Hong X. China's health reform update. Annual Review of Public Health. 2017 Mar 20;38:431-48. [↑](#footnote-ref-2)
3. Lehavi D, Barkol O, inventors; EntIT Software LLC, assignee. Regular expression matching. United States patent US 10,242,125. 2019 Mar 26. [↑](#footnote-ref-3)
